# Generating comprehensive functioning and disability data worldwide: development process, data analyses strategy and reliability of the WHO and World Bank Model Disability Survey

**DOI:** 10.1186/s13690-021-00769-z

**Published:** 2022-01-04

**Authors:** Carla Sabariego, Carolina Fellinghauer, Lindsay Lee, Kaloyan Kamenov, Aleksandra Posarac, Jerome Bickenbach, Nenad Kostanjsek, Somnath Chatterji, Alarcos Cieza

**Affiliations:** 1 Department of Health Sciences and Medicine, University of Lucerne, Lucerne, Switzerland; 2 Swiss Paraplegic Research, Nottwil, Switzerland; 3 Center for Rehabilitation in Global Health Systems, WHO Collaborating Center, University of Lucerne, Lucerne, Switzerland; 4 Sensory Functions, Disability and Rehabilitation, Department of Noncommunicable Diseases, World Health Organization, Avenue Appia 20, 1211 Geneva, Switzerland; 5 East Africa Human Development. The World Bank, Washington, DC 22304 USA; 6 Department of Classifications and Terminologies, World Health Organization, Geneva, Switzerland; 7 Department of Data and Analytics, World Health Organization, Geneva, Switzerland

**Keywords:** Model disability survey, Disability, Functioning, Reliability, International classification of functioning, disability and health, Rehabilitation

## Abstract

**Background:**

Data on functioning and disability collected at population level is essential to complement mortality and morbidity, to estimate rehabilitation needs of countries and regions and to monitor the Convention on the Rights of Persons with Disabilities (CRPD) and the Sustainable Development Goals (SDGs). The objective of this paper is to briefly report the development process of the WHO Model Disability Survey, its data analysis strategy as well as its reliability and ability to measure low to high levels of functioning and disability across countries.

**Methods:**

The development process is described in detail, and a secondary analysis using Rasch methods is conducted to report reliability and targeting using data from eight national and two regional implementations of the survey.

**Results:**

The currently available versions of the Model Disability Survey are presented. The survey has good to very good internal reliability and good targeting in all included countries.

**Conclusion:**

The participatory and evidence-based development, consideration of the expertise of stakeholders, the availability of previously developed ICF-based surveys, and WHO tools targeting functioning and disability are reflected in its good to very good psychometric properties. The survey has been implemented to date in Afghanistan, Cameroon, Chile, Costa Rica, India, Laos, Pakistan, Philippines, Sri Lanka, and Tajikistan, and is used to inform policy-making, to monitor the CRPD and SDGs and to plan the delivery of rehabilitation services.

**Supplementary Information:**

The online version contains supplementary material available at 10.1186/s13690-021-00769-z.

## Background

Data on functioning and disability collected at population level is essential to complement mortality and morbidity data and to estimate rehabilitation needs of countries and regions. Analyses using data from the Global Burden of Disease (GBD) Study – considering chronic respiratory and cardiovascular diseases, musculoskeletal, neurological, and mental disorders, neoplasms and sensory impairments – have shown that approximately 2.4 billion people worldwide could potentially benefit from rehabilitation at some point in their lives [1]. This staggering figure reflects current health and demographic trends, especially the continuing increase in the burden associated to non-communicable diseases (NCDs) and a rapid ageing of the global population [2, 3]. Both the GBD estimate and the fact that 15% of the world’s population experience severe disability [4], reiterate the need to collect national and regional comprehensive data on functioning and disability, using representative samples of the general population. Such data can guide stakeholders making decisions on the allocation of public resources to health, education, social protection, provision of rehabilitation services, etc. Moreover, these data allow the monitoring of the implementation of the United Nations Convention on the Rights of Persons with Disabilities and of the achievement of Sustainable Development Goals (SDGs).

The World Health Organization (WHO) and World Bank Model Disability Survey (MDS) is the current standard recommended by WHO to collect data on functioning and disability. The MDS is fully in line with the International Classification of Functioning, Disability and Health (ICF) [5] and collects data based on three key principles [6], namely that disability: 1) is a universal human experience, 2) is the outcome of the interaction between health conditions and contextual factors, and 3) is a matter of degree, ranging from low to high levels of severity. Given these principles, the MDS: 1) uses representative samples of the general population; 2) broadly considers the physical, human-built, attitudinal, and socio-political environment of specific populations, and 3) is suitable to compare persons with a range of health conditions experiencing mild, moderate, and severe levels of disability to persons who do not experience disability. Furthermore, a continuum ranging from no disability to extreme disability is estimated with MDS data, demonstrating the distribution of functioning and disability of a country.

Although several papers using MDS data, including the analyses of its metric properties [7] have been published so far, no paper has formally reported its participatory development process, the data analyses strategies, and metric properties observed across countries. Countries generally obtain this information on request from WHO, but there have been recurrent calls for a scientific manuscript that can be referred to in national and international publications. The objective of this paper is therefore to briefly report the development process of the MDS, its data analyses strategy as well as its reliability and ability to measure low to high levels of functioning and disability across countries.

## Methods

### Development process

**Fig. 1:**
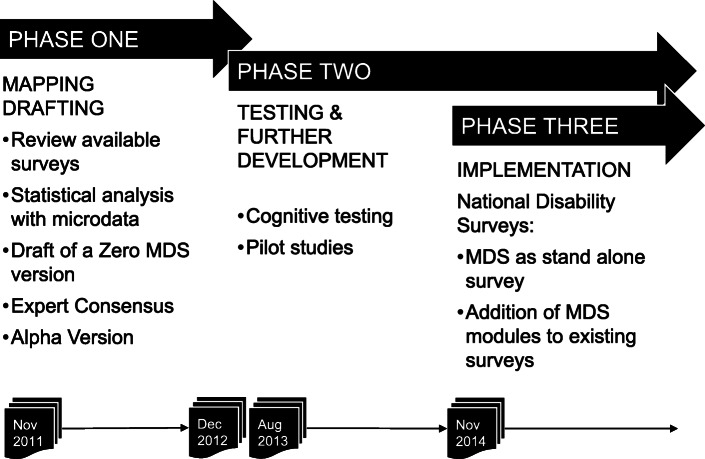
Development process of the Model Disability Survey

WHO initiated the development of the MDS in 2011, right after the publication of the WHO World Report on Disability (WRD), to fill a gap: a scarcity of standardized ICF-based surveys designed to collect comprehensive information on functioning and disability. The development process (Fig. 1) was structured, planned, and overseen by a Steering Committee consisting of WHO staff, World Bank staff and external consultants, and consisted of a preparatory and a testing phase, described in detail below.

#### Preparatory phase

The preparatory phase lasted from November 2011 to December 2012 and had as a goal the participatory development of an alpha version of the MDS for field testing. The specific aims were to a) draft a very first “zero” version of the MDS, learning from available health and disability surveys; b) prepare and conduct a large expert consensus meeting to obtain feedback on the “zero” version and c) propose an alpha version for the testing phase, informed both by available surveys and by expert feedback.

In a first step, health and disability surveys conducted worldwide since the endorsement of the ICF in 2001 were identified for every country listed in the Technical Appendix A of the WRD [4]. Medline, as well as webpages of statistical offices, health ministries and relevant organizations were searched, and questionnaires, detailed descriptions, and micro data from 100 of the 179 identified surveys were recorded. Using pre-defined criteria, 48 surveys were selected for in-depth content analysis of questions and response options used to measure functioning and disability, using the ICF as a reference framework. For twelve surveys^1^ in which micro data was available, Rasch analyses [8] of functioning and disability data were carried out (for each survey separately) to understand the impact of disability conceptualization, survey content, as well as wording, framing and response options of questions on the prevalence estimates of disability (Survey-specific reports are available from WHO on request). Rasch analysis [8–10] was also used to evaluate psychometric properties (reliability, targeting, fit, monotonicity, stochastic ordering, local item independence, dimensionality, and differential item functioning) and to identify questions suitable to discriminate different degrees of disability (mild, moderate, severe). The analyses showed that disability surveys were generally designed for persons with rather high levels of disability, using for instance filters to pre-select the sample. Negative implications of this approach were extensively discussed elsewhere [7]. Additionally, the content of three WHO tools was mapped and compared, using the ICF as a reference framework: WHO World Health Survey [11], the WHO Study on global AGEing and adult health (SAGE) [12] and the WHO Disability Assessment Schedule (WHODAS) [13].

All ICF domains and categories identified in the WHO tools and in the health and disability surveys were included in the very first “zero” version of the MDS, namely: seeing, hearing, pain, energy and drive, breathing, affect, cognition, mobility, self-care, interpersonal relationships, handling stress, carrying out daily routine, communication, household tasks, work & schooling, recreation, leisure and community participation, caring for others and citizenship. In the operationalization of modules and questions, the inclusion of items capturing low, moderate, and high levels of disability in a balanced way was strived for and informed by the Rasch analyses of identified surveys. Examples of items capturing low levels of disability are the ones targeting vigorous activities, cutting toenails or forgetfulness; examples targeting high levels of disability include eating, toileting and forgetting important things in daily life. To conform with two selections of ICF categories considered the minimum information of functioning to be collected at population level, namely the minimal generic ICF set [14] and the ICF rehabilitation core set [15], questions about changing body position, standing, using transportation, looking after one’s health and relationships with friends were also included in the “zero” version. For all other MDS modules, either modules of existing WHO surveys, for instance, the responsiveness of health system module used in a range of WHO surveys, or validated questionnaires, for instance the Attitudes to Disability Scale [16], were used. The exact list of all tools included in the MDS modules is described in detail in the MDS manual, which is available online [17].

The “zero” version was presented in the expert meeting held in December 5th and 6th 2012, at the WHO headquarters in Geneva, which brought together 28 international stakeholders from 20 countries, who represented national statistical offices, health ministries, Disabled Persons Organizations (DPO), the Washington City Group on Disability Statistics (WG), United Nations (UN) agencies whose mandate included disability data i.e. UN International Children’s Emergency Fund (UNICEF), disability and development organizations, academic and research organizations as well as donors for whom disability data was a priority. The feedback received in this meeting informed the finalization of the MDS alpha version for the testing phase.

#### Testing phase

The testing phase started in August 2013, lasted approximately 1 year, and comprised both cognitive testing to identify problems with the formulation and understanding of questions operationalizing specific areas of functioning, for instance eating or toileting, and field testing to test the questionnaire as a whole, for instance skip patterns and the order of the modules, and to identify issues, for instance culturally sensitive questions. The survey was revised and improved after each cognitive and field-testing round.

Three rounds of cognitive testing were carried out in five countries: the United States of America (US), Cambodia, Malawi, Nepal, and China. Cognitive testing was conducted on request of WHO by the US National Center for Health Statistics, by the Institute for Survey Research at the University of Michigan and by Statistics Norway. All cognitive testing was supervised by WHO and collaborating partners were requested to use a manual adapted by colleagues of the University of Michigan for the World Mental Health Study [18]. The “think aloud” approach was used, and a cognitive testing protocol was submitted to WHO for approval before the test started.

Field tests were carried out in Cambodia by Statistics Norway, the National Institute of Statistics Cambodia and WHO, including 500 adults and 500 children (August 2014); in Malawi by Statistics Norway, including 500 adults and 500 children (March 2015); in Pakistan by the Ministry of Health, Pakistan Bait-ul-Mal and WHO, including 3977 adults (February and March 2015); and in Oman by the Ministry of Health and WHO, including 300 adults and 300 children (October and November 2015). All field tests worked with purposive sampling to obtain a balanced number of interviews from males and females, persons of different age groups, and persons with and without health conditions. The samples were also context specific and defined by the office for statistics of the country. For instance, in Cambodia a quota sample was used following a stratification by age, sex and education, in urban and rural areas, and targeting the inclusion of healthy respondents as well as persons with impairments and health conditions to test the feasibility and validity of the MDS in each of these groups [19].

### Data analysis strategy

Data analyses follow a corresponding MDS manual, which includes R codes [20, 21] is available on request from WHO.

In the MDS, functioning and disability are understood as a matter of degree, a continuum, ranging from low to high levels of severity. Moreover, two perspectives are considered: intrinsic capacity – defined as all the physical and mental capabilities of an individual given existing health conditions – and performance – defined as the outcome of the interaction between the individual’s intrinsic capacity and facilitating or hindering features of the physical, human-built, attitudinal, and socio-political environment. This definition of performance corresponds to the WHO understanding of functioning and disability.

In line with the approach described above, the originally ordinal-scaled MDS questions are used to develop metrical scales of capacity (Module 5000) and performance (Module 4000) using Rasch analysis. The distribution of the adjusted disability metrics in a population is then partitioned according to pre-defined cut-offs (Table 1) and persons experiencing no, mild, moderate, and severe disability can be identified. These groups are used to determine the prevalence of severe disability in a population and to disaggregate survey indicators by disability level, including direct comparisons to persons who do not experience disability.

**Table 1:** Cut-offs used to identify persons with mild, moderate, and severe disability. “Score” refers to the performance metric score. “Mean” refers to sample mean of the given score. “1SD” refers to one standard deviation of the given score

Level of disability	Cut-off
**No**	Score < Mean – 1SD or Score = 0
**Mild**	Mean – 1SD < Score < Mean
**Moderate**	Mean < Score < Mean + 1SD
**Severe**	Score ≥ Mean + 1SD

#### Developing metrical scales

The partial credit model (PCM) or the polytomous Rasch model is used to develop the scales with metrical properties [9]. The PCM is a model from the Rasch family that allows to test if core measurement assumptions [10] are fulfilled and the ordered categorical (polytomous) items can be summed to obtain an interval-scaled latent score. Both persons and items (questions) can be located on a continuous latent construct metric. For each respondent, the person ability, i.e., the location of the person on the continuum, and for each item, the item difficulty, i.e., the location of a question on the same continuum are estimated. Additionally, difficulty thresholds are estimated for the response options at each item. Item thresholds represent the location on the measurement continuum where two adjacent response options, for instance 2 (mild) and 3 (moderate), are equally likely to occur. The continuous latent construct metric interval units are logits. Logits represent the relative frequency of endorsing a response option versus not endorsing it and allow one to determine – in the case of the MDS with high scores indicating higher disability – if a person has a high level of functioning (negative logit) or a low level of functioning (positive logit). The residuals of the Rasch analysis allow for the calculation of the items’ fit [22], the dimensionality of the scale [23], the presence of local item dependencies [24], and potential differential item functioning [25]. The person ability scores, and their measurement precision allow one to calculate the Person Separation Index (PSI), an indicator for the internal reliability of the scale, which is based on the distribution of the person ability and their measurement error. The PSI ranges from 0 to 1 and describes the part of the ability estimates’ variance that is not attributable to measurement error. PSI values of 0.7 indicate acceptable reliability for population-level measurement while for individual measurement PSI > 0.85 would be expected [10]. The logit ability estimates are linearly transformed to obtain a more user-friendly score range from 0 (lowest level of disability) to 100 (highest level of disability).

**Fig. 2:**
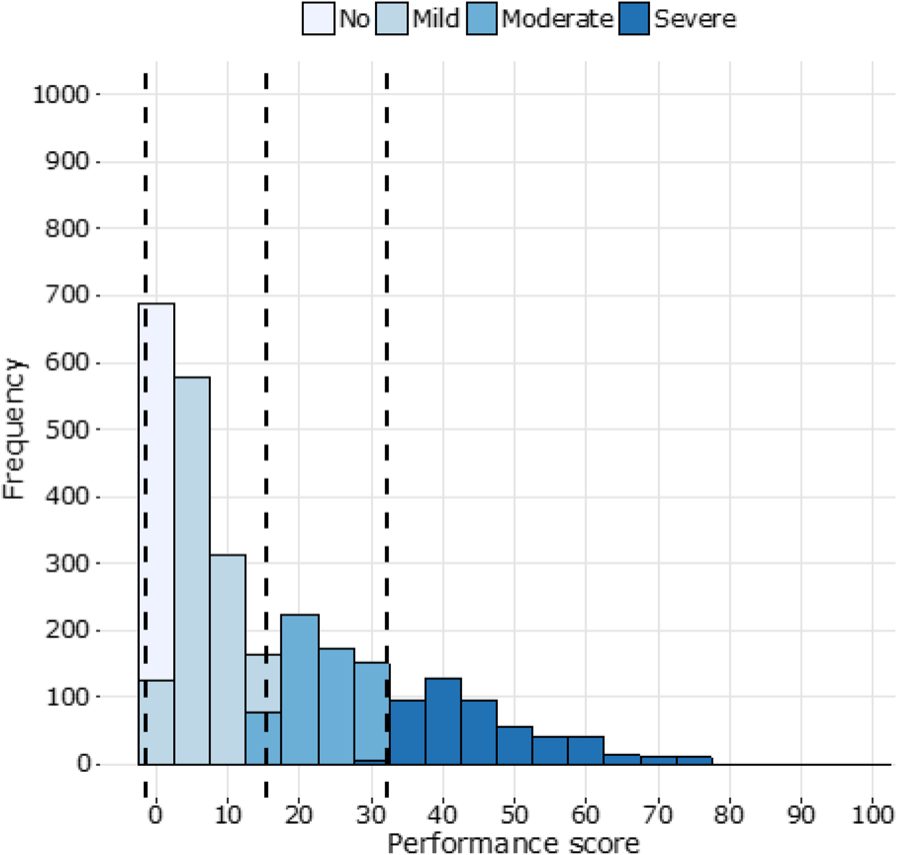
Example of the performance metric and distribution of the population on the continuum ranging from 0 (lowest level of problems) to 100 (highest levels of problems). No, mild, moderate, and severe are the defined disability levels using pre-defined thresholds (separated by the dashed lines)

To ensure that the final performance scores reflect indeed the interaction between health conditions and the environment, random forest analysis [26] is used to obtain adjusted performance scores. This analysis sets the Rasch-based person ability estimates for the performance scale (Module 4000) as depending on the Rasch-based capacity scores and environmental factors, which include among others personal assistance, assistive devices, social support, attitudes of others (see Table 2). The adjusted performance values predicted by the model are then used to obtain the disability distribution (Fig. 2). This distribution shows the frequency (Y-axis) of observed performance scores grouped into score intervals (X-axis) in the survey population and reflects how people function in daily life given their capacity level and the environmental barriers and facilitators that shape their lives. The dashed vertical lines indicate the cut-offs used for no, mild, moderate, and severe disability (Table 2).

**Table 2:** Characteristics of the two Model Disability Survey (MDS) versions; modules in bold are mandatory

Name	MDS	Brief MDS
**Webpage**	https://www.who.int/activities/collection-of-data-on-disability	
**Developers**	World Bank and WHO	
**Conceptual framework**	International Classification of Functioning, Disability and Health (ICF)	
**Definition of disability**	Disability is defined as the outcome of the interaction between a person with a health condition and the physical, human-built, attitudinal, and socio-political environment in which the person lives, and as a continuum, ranging from low to high levels of disability.	
**Year**	Project initiated in Dec 2011; first version proposed in Dec 2012	First version proposed in August 2016
**Type of instrument**	Household stand-alone survey	Module to be integrated in household surveys, such as health or living conditions and standards surveys
**Sample**	Representative sample of the general population (country or region)	Depends on the household survey used
**Type of data**	Self-report data obtained through an interview by trained interviewers	
**Modules**	1000 Socio-demographic characteristics 2000 Work history and benefits **3000 Environmental factors** **4000 Functioning** **5000 Health conditions and capacity** 6000 Health care utilization 7000 Well-being 8000 Empowerment Optional children module	Brief versions of: **3000 Environmental factors** **4000 Functioning** **5000 Health conditions and capacity**
**Questionnaires**	Household and individual questionnaire	Module
**Selection of respondents**	A household member 18 years old or older is randomly selected for the individual questionnaire; no filters are applied.	Depends on the household survey used; no filters are applied.
**Implementation**	Recommended every 5 to 10 years	Continuous implementation (at the schedule of the main survey)
**Objectives**	- determine the current distribution of disability in a population; - estimate the prevalence of severe, moderate and mild disability; - identify unmet needs of persons with different levels of disability; - identify what are the barriers and inequalities faced by persons with different levels of disability.	- continuous monitoring of the distribution of disability in populations; - disaggregation of indicators by disability for monitoring of purposes.
**Cut-offs**	Recommended cut-offs applied to identify persons with none, mild, moderate, and severe levels of disability

The metric analyses with the Rasch model of all full MDS implementations so far (Afghanistan, Cameroon, Chile, Costa Rica, India, Laos, Pakistan, Philippines, Sri Lanka, and Tajikistan) has supported the good psychometric properties of the MDS as a tool to measure functioning and disability. The metric analyses are part of the official country reports, available on request from WHO (conditional on country approval). An illustrative metric analysis as described above is reported in detail elsewhere [7].

#### Identifying persons with no, mild, moderate, and severe disability for data disaggregation

The assessment of specific needs of people experiencing different levels of disability, the identification of inequalities and barriers they face, and the disaggregation of indicators by disability levels are essential for informing policy-making. Therefore, it is necessary to define cut-offs to partition the disability continua and identify who are the persons experiencing mild, moderate, and severe levels of disability. WHO’s current suggested cut-offs are based on the distribution of the sample, using the mean and standard deviation of the disability scores (Table 1). It is important to stress that for the moment cut-offs are country-specific and dependent on the distribution of disability in the country.

### Reliability and targeting

It is recommended to implement the MDS in regular intervals to monitor trends over time in the disability distribution of specific populations. The reliability of the capacity and performance metrics is therefore especially relevant to ensure precision, replicability, and consistency from one data collection wave to another.

As mentioned above, the PSI is the estimate of internal consistency (reliability). Additionally, a scale with good “targeting” should include questions suitable to measure the ability of persons with low, moderate, and high levels of functioning or disability. A scale has a good targeting when the items used for measurement (questions) match the ability of all respondents, including respondents with low, moderate, and high ability (ability is in our case the level of functioning or disability). Ideally, the mean difficulty of a scale should approximate the mean level of ability of the target population, and the spread of the difficulty thresholds should encompass the population’s ability range. Technically, as the PCM estimation centers the difficulty of the items to be zero, the derived mean ability estimates should also be close to zero. In general, less than half a logit difference from zero is tolerable and would still indicate a good centering. A difference of half a logit or larger is suggested as clinically meaningful in polytomous response scales [27].

The MDS has been implemented since 2014 by a range of countries worldwide with technical support from WHO. In this article, we carry out a secondary analysis to report reliability and targeting using data from national implementations in Chile (2014), Sri Lanka (2015), the Philippines (2017), Costa Rica (2018) and Afghanistan (2019), from regional implementations in the Adamawa region in Cameroon (2016) and in Balochistan, Pakistan (2017) as well as national implementations of the brief MDS (see Table 2) in India, Laos, and Tajikistan in 2018. The sample size indication in the results section relates to the number of cases available to calibrate the capacity and performance scales (Tables 3 and 4).

**Table 3:** Reliability (PSI) and targeting (item difficulty and person ability distribution, and difficulty range) of the capacity scale by country. PSI: Person separation index; Mean (SD): Mean and standard deviation

Country (region)	Coverage	Year	Sample Size	PSI	Item Difficulty Mean (SD)	Person Ability Mean (SD)	Difficulty Range Thresholds min; max
**Afghanistan**	National	2019	14196	0.89	0.41 (1.016)	−1.69 (1.24)	− 1.79; 2.18
**Cameroon, Adamawa**	Regional	2016	559	0.89	0.58 (2.01)	−2.08 (1.71)	−3.57; 3.61
**Chile**	National	2014	12265	0.82	0.25 (1.28)	−1.99 (1.44)	−3.04; 2.12
**Costa Rica**	National	2018	2390	0.83	0.06 (1.03)	−1.75 (1.12)	−2.41; 2.1
**India**	National (brief MDS)	2018	2997	0.89	0.21 (0.68)	−0.88 (1.24)	−1.1; 1.47
**Laos**	National (brief MDS)	2018	2461	0.89	0.28 (0.74)	−0.69 (1.08)	−1.16; 1.7
**Pakistan, Balochistan**	Regional	2017	451	0.94	0.5 (1.27)	−1.08 (1.56)	−2.26; 3.47
**Philippines**	National	2017	10246	0.86	0.45 (1.34)	−2.36 (1.42)	−3.18; 4.72
**Sri Lanka**	National	2015	3000	0.84	0.21 (1.21)	−1.42 (1.35)	−2.45; 2.25
**Tajikistan**	National (brief MDS)	2018	2999	0.83	0.27 (1.05)	−1.25 (1.4)	−1.63; 2.20

**Table 4:** Reliability (PSI) and targeting (item difficulty and person ability distribution, and difficulty range) of the performance scale by country. PSI: Person separation index; Mean (SD): Mean and standard deviation

Country	Coverage	Year	Sample Size	PSI	Item Difficulty Mean (SD)	Person Ability Mean (SD)	Difficulty Range Thresholds min; max
**Afghanistan**	National	2019	14130	0.91	0.29 (0.9)	−1.38 (1)	− 1.81; 2.85
**Cameroon, Adamawa**	Regional	2016	559	0.88	0.20 (0.86)	− 0.74 (0.72)	− 1.46; 2.02
**Chile**	National	2014	12265	0.87	0.23 (0.69)	−1.06 (0.87)	− 1.47; 2.34
**Costa Rica**	National	2018	2390	0.87	0.21 (0.91)	−1.18 (0.83)	−1.68; 3.54
**India**	National (brief MDS)	2018	2981	0.91	0.17 (0.53)	−0.64 (1.32)	−0.73; 1.57
**Laos**	National (brief MDS)	2018	2410	0.89	0.23 (0.72)	−0.64 (1.04)	−1.24; 1.47
**Pakistan, Balochistan**	Regional	2017	282	0.93	0.95 (1.94)	−0.46 (1.42)	−3.56; 4.62
**Philippines**	National	2017	10247	0.88	0.52 (1.22)	−1.44 (1.17)	−2.33; 3.09
**Sri Lanka**	National	2015	3000	0.88	0.17 (0.76)	−1.11 (0.97)	−1.77; 2.17
**Tajikistan**	National (brief MDS)	2018	2992	0.83	0.26 (0.97)	−1.59 (1.21)	−1.31; 1.81

## Results

Two versions of the MDS are currently available online: a long, standalone household survey for adults, and a brief version, developed to be integrated in health and other population surveys to facilitate easier and straightforward, continuous monitoring of functioning and disability in a region or a country [17]. The characteristics of both versions are summarized in Table 2. The development and metric properties of the brief MDS are reported elsewhere [28]. A children module of the MDS is as well available and includes capacity and performance modules, and questions on health conditions and environmental factors.

All modules of the MDS are described in detail in the MDS manual, which is available online [17], and a brief summary is presented in the supplementary material.

The reliability of the MDS capacity scale across 10 countries where the MDS survey (standalone and brief) was conducted is shown in Table 3 and ranged from 0.82 to 0.94, from good to very good. The highest reliability was found in Pakistan (PSI = 0.94) and the lowest in Chile (PSI = 0.82). With one exception, i.e., Cameroon with a mean item difficulty of 0.58 (SD = 2.01), the mean difficulty of the capacity items for the countries were less than half a logit away from zero. Costa Rica showed the best centering of the item difficulty estimates (mean = 0.06, SD = 1.03). The person ability estimates for the capacity scale are all under zero and all more than half a logit away from zero. This indicates that in general the populations had less problems in capacity than what the scale would target in average. The lowest mean capacity is found for the Philippines with a mean ability of − 2.26 logits (SD = 1.42) and the closest to zero is found for Laos with a mean ability of − 0.69 (SD = 1.08).

Table 4 shows the results of the metric analysis with the performance scale of the standalone or the brief version of the MDS survey. The sample size indication relates to the number of cases available to calibrate the performance metric and can differ from the sample sizes of the capacity metric. The sample sizes ranged from *N* = 282 (Pakistan) to *N* = 14,130 (Afghanistan). The reliability of the MDS performance scale across 10 countries where the MDS survey (standalone and brief) was conducted ranged from 0.83 to 0.93, from good to very good. As for the capacity scale, the highest reliability was found in Pakistan, Balochistan (PSI = 0.93) and the lowest in Tajikistan (PSI = 0.83). Sri Lanka showed the best centering of the item difficulty estimates (mean = 0.17, SD = 0.76). Except for the MDS survey in Pakistan, Balochistan (mean 0.95, SD = 1.94) and Philippines (mean = 0.52, SD = 1.22) the mean difficulties of the MDS performance items were less than half a logit away from zero. The person ability estimates for the performance scale are all under zero and indicate that the mean level of performance in the populations was higher than what the performance scale would target in average. The lowest mean level of performance is found for Tajikistan with a mean ability of − 1.59 logits (SD = 1.21) and the closest to zero is found for Pakistan with a mean ability of − 0.46 (SD = 1.42).

The negative ability estimates (mean person ability in Tables 3 and 4) in the analysis of the performance and capacity scales consistently indicate that the average functioning of the participants is higher than what the scales target on average (mean item difficulty in Tables 3 and 4). This is a reflection of using a representative sample of the general population in the MDS: the scale is constructed to measure a continuum ranging from low to high levels of disability or functioning (difficulty range thresholds in Tables 3 and 4), but the majority of the population usually has either no or low levels of disability. This is neither surprising nor a problem since the general population sample was consciously selected to allow for the observation of trends over time in the prevalence of persons with mild, moderate and severe disability as well as for the direct comparison of the life situation of persons in those groups, for instance regarding employment rates, with the life situation of persons not experiencing disability or functioning limitations. The advantages of using a representative sample of the general population have been discussed in detail elsewhere [7].

## Discussion

In this article, we briefly reported the development process of the MDS, its data analysis strategy as well as its internal reliability and ability to measure low to high levels of disability across countries. The MDS is currently available as a comprehensive standalone household survey for adults and as a brief version suitable to be integrated in population surveys, mainly health surveys, to enable a continuous monitoring of functioning at regional and national levels. The reliability as well as the ability of the MDS to measure the functioning of persons with low, moderate, and high levels of disability is good to very good across a range of countries in different world regions. Variations across countries are mostly due to cultural differences such as living context, economic situation, religion, or the education system. The MDS has so far been implemented in Afghanistan, Cameroon, Chile, Costa Rica, India, Laos, Pakistan, Philippines, Sri Lanka, and Tajikistan, and is the current WHO standard for measuring functioning and disability to inform policy-making in countries and regions, to monitor the CRPD and SDGs as well as to plan and delivery rehabilitation to the population in need. WHO data collection instruments like the World Health Survey [13], WHO SAGE [14] or the WHODAS [15] undergo generally long and complex developments. However, development processes are rarely published in peer review journals and remain not accessible to stakeholders that do not use their implementation manuals. Given the importance of the MDS for the CRPD and SDGs as well as for supporting the strengthening of rehabilitation in health systems, we aimed to make its development process transparent and accessible to a broader range of stakeholders.

Health and disability surveys are the most comprehensive way of gathering information about functioning and disability at population level. Their major goal is to support the evidence-informed development and monitoring of policies and strategies to improve the health and societal participation of persons with health conditions. A range of health and disability surveys were available before the MDS, but disability rates estimated with them varied substantially and inconsistently between countries, for instance ranging from 3% in Algeria to 32.2% in Finland [4]. These variations were due the fact that “[ …] *measures of disability vary according to the purpose and application of the data, the conception of disability, the aspects of disability examined [ …*], *the definitions, question design, reporting sources, data collection methods, and expectations of functioning*” ([4], page 21). The WRD stressed the lack of standardized survey instruments developed to collect comprehensive and relevant information for disability policy and rehabilitation planning, suitable for international comparisons and that could provide the data needed by countries to accomplish their obligation under Article 31 of the CRPD [4] and to monitor SDGs. The MDS is the WHO answer to fill this gap [6].

Comprehensive population data on functioning is the basis for planning and delivering rehabilitation services as well as for monitoring the impact of integrating rehabilitation into health systems. The relevance of rehabilitation has been recently underpinned by global need estimates based on the Global Burden of Disease study 2019 [1]. Authors showed that globally, in 2019, ca. 2.4 billion persons had conditions that would benefit from rehabilitation, which represents ca. 30% of the world’s population and supports the definition of rehabilitation as a public health strategy. Since optimizing functioning is the core goal of rehabilitation, data on functioning at all levels is urgently needed. Data at population level, including detailed information on functioning and its determinants (health conditions and contextual factors), is key for supporting rehabilitation policy and the development of services matched to population needs, as well as to monitor health trends over time, especially the impact of scaling up rehabilitation in countries and regions [29, 30]. The MDS is therefore very well-suited to support member states that are following the WHO 2030 Call for Action [30] and strengthening rehabilitation in their health systems to plan and monitor the impact of their efforts.

Our work must be understood in light of its limitations. First, a WHO survey development process is not a scientific project, and the use of standard sections of research manuscripts (introduction, methods, results, and discussion) lead to a relatively unusual reporting of data. Nevertheless, we consider the manuscript relevant to stakeholders and to the scientific community. Secondly, we restricted the presentation of psychometric properties to two key aspects, reliability and targeting, although they are much broader than these two aspects. We nevertheless considered them the most relevant ones for this publication, and broad psychometric evaluations of the MDS have been already published, as cited in the manuscript. Thirdly, WHO supports every country implementing the MDS and each data collection and analysis is by definition country specific. Universal MDS metrics of capacity and performance would be ideal for direct comparisons across countries, but this is a challenging endeavor that would require data from considerably more countries than available so far. Lastly, a key and challenging issue of using a general population sample is defining the cut-offs for identifying persons with no, mild, moderate and severe disability. There is no perfect cut-off. When selecting cut-offs for the MDS, we looked for a cut-off recommendation that is fit for the MDS purpose. Several approaches have been considered, including the health state-based cut-off used in the WRD (mean score of persons reporting at least extreme difficulties in at least one of eight functioning domains as well as persons reporting at least one of four chronic conditions likely to lead to disability) and a range of statistical cut-offs. Since each data collection and data analyses is country specific, we decided to recommend a statistical cut-off so that at least percentages of persons in the groups of persons with mild, moderate and severe disability could be compared across countries. The cut-off presented in this paper is nevertheless a recommendation and countries might choose to use different cut-offs.

## Conclusion

The participatory and evidence-based development, consideration of the expertise of stakeholders, the availability of previously-developed ICF-based surveys, and WHO tools targeting functioning and disability are reflected in MDS' good to very good psychometric properties. The survey has been implemented to date in Afghanistan, Cameroon, Chile, Costa Rica, India, Laos, Pakistan, Philippines, Sri Lanka, and Tajikistan, and is used to inform policy-making, to monitor the CRPD and SDGs and to plan the delivery of rehabilitation services.

## Supplementary Information

**Additional file 1.**

## Data Availability

Data are available from the authors upon request.

## Abbreviations

GBD: Global Burden of Disease
NCDs: Non-communicable diseases
CRPD: Convention on the Rights of Persons with Disabilities
SDGs: Sustainable Development Goals
WHO: World Health Organization
MDS: Model Disability Survey
ICF: International Classification of Functioning, Disability and Health
WRD: World Report on Disability
SAGE: Study on global AGEing and adult health
DPO: Disabled Persons Organizations
WG: Washington City Group on Disability Statistics
UN: United Nations
UNICEF: United Nations International Children’s Emergency Fund
US: United States of America
PCM: Partial Credit Model
PSI: Person Separation Index

## Footnotes

1 Finland: Health 2000; Spain: EDAD2008; Canada: PALS; Chile: ENDISC; Nicaragua: ENDIS; Lesotho & Swaziland: Living conditions among people with disabilities; Tanzania: Tanzania Disability Survey; Morocco: National Disability Survey; Oman; New Zealand: Disability counts 2001; Kazakhstan, Maldives, Sri Lanka, Cambodia, Mongolia, Philippines: WHO & ESCAP Disability Survey, Pilot 2009.
