# Supplementary material for: Generating comprehensive functioning and disability data worldwide: development process, data analyses strategy and reliability of the WHO and World Bank Model Disability Survey
Source: Arch Public Health. 2022 Jan 4;80:6. doi: 10.1186/s13690-021-00769-z (PMC8725367; doi:10.1186/s13690-021-00769-z)
Supplement: Supplementary file 1 — Additional file 1. [file 13690_2021_769_MOESM1_ESM.docx]

**Supplementary material 1**

Modules of the individual questionnaire of the MDS.

| **Module** | **Name** | **Brief content description** |
| --- | --- | --- |
| **1000** | Socio-demographic | Provides information on socio-economic indicators, such as educational level, marital status or income. |
| **2000** | Work history and benefits | Assesses detailed information on work, such as whether the respondent has ever worked for pay, type of work, place of work and for how long the respondent has worked. Age at which the respondent started or stopped working and reasons for stopping working are targeted. |
| **3000*** | Environmental factors | Contains a broad inventory of questions about:  - Hindering or facilitating aspects of the general environment  - Use and need for personal assistance  - Family and social support  - Attitudes of others  - Accessibility to information  - Regular use of medication  - Use and need for assistive products for self-care, mobility, cognition, seeing and hearing  - Presence and need of modifications at home, school, work, and community. |
| **4000*** | Functioning | Comprises questions targeting performance, i.e., how people function in multiple domains given their intrinsic capacity and the environmental barriers and facilitators that shape their life experiences.  The original questionnaire includes 47 questions covering the extent of problems in the following 17 functioning domains:   - mobility - hand and arm use - self-care - seeing - hearing - pain - sleep - energy - breathing - affect - interpersonal relationships - handling stress - communication- - cognition - household tasks - community - citizenship participation - caring for others - work and schooling |
| **5000*** | Health conditions and capacity | Comprises questions targeting both the presence of health conditions and difficulties in capacity, defined as the synthesis of all intrinsic physical and mental capacities of a person, determined by his or her health conditions, health decrements or impairments.  Altogether 17 capacity questions cover the same 17 domains of Module 4000 and target the extent of difficulties.  Additionally, a self-report part about 26 health conditions and impairments is included. For each health condition or impairment, the respondent is asked “Do you have [disease name]?”. If a respondent responds “yes” to the entrance question, three questions follow: 1) whether any doctor or any other health professional has ever told them that they have the health condition or problem; 2) whether he or she has been given any medication for the health condition or problem in the last 12 months; and 3) whether he or she has been given any other kind of treatment, beyond medicines, for the health conditions or problem in the last 12 months. |
| **6000** | Health care utilization | Standard module of WHO developed to assess the responsiveness of the health care system to the respondent's health care needs. |
| **7000** | Well-being | Covers the respondent's thoughts about his or her own life and well-being, and includes questions about quality of life, loneliness, and subjective well-being. |
| **8000** | Empowerment | Covers aspects of empowerment, such as the sense of having control about the own life and to what extend persons feel confident to overcome problems even in difficult situations. |

Modules of the brief version of the MDS.

| **Module** | **Name** | **Brief content description** |
| --- | --- | --- |
| **3000*** | Environmental factors | Environmental factors are measured in the Brief MDS in two sections.  Module “3000A Environmental Factors” is placed at the beginning of the Brief MDS and encompasses nine questions about hindering or facilitating aspects of the environment, family and social support, and attitudes of others. Module “3000B Personal Assistance and Assistive Products” is placed at the end of the Brief MDS and includes specific questions that are generally relevant only to the part of a sample experiencing higher levels of disability: use and need of personal assistance and assistive products. |
| **4000*** | Functioning | Comprises questions targeting performance, i.e., how people function in multiple domains given their intrinsic capacity and the environmental barriers and facilitators that shape their life experiences.  Performance is measured in the Brief MDS using 12 questions asking how much of a problem the person experiences in the following domains:   - Mobility (walking a kilometre, getting where you want to go and using public or private transportation); - Self-Care (being clean and dressed, toileting and looking after your health); - Energy and drive (feeling tired and not having enough energy); - Dealing with stress (coping with all the things one must do); - Cognition (remembering important things in day-to-day life); - Household tasks (getting household tasks done); - Community participation (joining community activities); - Work and education (getting things done as required at work or school). |
| **5000*** | Health conditions and capacity | Capacity is measured in this module using 12 questions asking how much difficulty the person has because of a health condition in:   - Seeing (at a distance) - Hearing - Mobility (walking or climbing steps) - Cognition (remembering or concentrating) - Self-care (washing or dressing) - Energy and drive (sleeping) - Household tasks - Community participation (joining community activities) - Affect (feeling sad, low, worried or anxious) - Interpersonal relationships (getting along with close people) - Pain   This section starts with the question “How would you rate your health today?” to support respondents focusing their attention on health. The last question of this module asks about the presence of a health condition. This question must not be added to a health survey. |

Modules of the children version of the MDS.

| **Module** | **Name** | **Brief content description** |
| --- | --- | --- |
| **H2001 to H2008** | Personal support, assistive devices, or medication | Comprises questions that are intended to capture any personal support, assistive devices, or medication a child might receive or use.  The answers given to these questions are essential for all the following questions about problems the child might experience (starting with H2009). |
| **H2009 to H2034** | Functioning | Comprises questions that indicate problems the child may have because of his or her health taking into account the positive or negative influence of any personal support, assistive devices or medication the child might receive or use.  Example: If a child has a hearing impairment but uses a hearing aid that makes hearing not a problem, then the expected answer to the corresponding question is “no problem”.  In some questions, the expected ability of children of the same age should be used by the respondent as a reference to judge whether the child has problems or not.  Problems are characterized by not getting things done in the way he or she wants to or not getting them done at all and include:   - Seeing - Hearing - Pain - Energy and drive - Breathing - Affect - Self-Care - Behaviour - Communication - Learning - Coping with change - School - Playing - Community Life |
| **H2035a to H2058a** | Health conditions | In this part respondents are first asked about the presence of health conditions. In a second step, questions about diagnosis and treatment are stated only for the endorsed health conditions. |
| **H2059 to H2066** | Capacity | Comprises questions that indicate difficulties the child may have only because of his or her health (health conditions or further health problems)  The expected ability of children of the same age are used by the respondent as a reference to judge whether the child has difficulties or not.  Difficulties are characterized by not getting things done in the way he or she wants to or not getting them done at all.  Respondents must NOT take into account any medicines, personal assistance, aids or modifications that might make the difficulties better or worse. |
